# Using high-repeatable radiomic features improves the cross-institutional generalization of prognostic model in esophageal squamous cell cancer receiving definitive chemoradiotherapy

**DOI:** 10.1186/s13244-024-01816-3

**Published:** 2024-10-07

**Authors:** Jie Gong, Qifeng Wang, Jie Li, Zhi Yang, Jiang Zhang, Xinzhi Teng, Hongfei Sun, Jing Cai, Lina Zhao

**Affiliations:** 1 Department of Radiation Oncology, Xijing Hospital, Fourth Military Medical University, Xi’an, China; 2 Department of Radiation Oncology, Sichuan Cancer Hospital & Institute, Sichuan Cancer Center, School of Medicine, University of Electronic Science and Technology of China, Chengdu, China; 3 Department of Health Technology and Informatics, The Hong Kong Polytechnic University, Hong Kong, China

**Keywords:** Esophageal cancer, Radiomics, Repeatability, Local recurrence-free survival, Overall survival

## Abstract

**Objectives:**

Repeatability is crucial for ensuring the generalizability and clinical utility of radiomics-based prognostic models. This study aims to investigate the repeatability of radiomic feature (RF) and its impact on the cross-institutional generalizability of the prognostic model for predicting local recurrence-free survival (LRFS) and overall survival (OS) in esophageal squamous cell cancer (ESCC) receiving definitive (chemo) radiotherapy (dCRT).

**Methods:**

Nine hundred and twelve patients from two hospitals were included as training and external validation sets, respectively. Image perturbations were applied to contrast-enhanced computed tomography to generate perturbed images. Six thousand five hundred ten RFs from different feature types, bin widths, and filters were extracted from the original and perturbed images separately to evaluate RF repeatability by intraclass correlation coefficient (ICC). The high-repeatable and low-repeatable RF groups grouped by the median ICC were further analyzed separately by feature selection and multivariate Cox proportional hazards regression model for predicting LRFS and OS.

**Results:**

First-order statistical features were more repeatable than texture features (median ICC: 0.70 vs 0.42–0.62). RFs from LoG had better repeatability than that of wavelet (median ICC: 0.70–0.84 vs 0.14–0.64). Features with smaller bin widths had higher repeatability (median ICC of 8–128: 0.65–0.47). For both LRFS and OS, the performance of the models based on high- and low-repeatable RFs remained stable in the training set with similar C-index (LRFS: 0.65 vs 0.67, *p* = 0.958; OS: 0.64 vs 0.65, *p* = 0.651), while the performance of the model based on the low-repeatable group was significantly lower than that based on the high-repeatable group in the external validation set (LRFS: 0.61 vs 0.67, *p* = 0.013; OS: 0.56 vs 0.63, *p* = 0.013).

**Conclusions:**

Applying high-repeatable RFs in modeling could safeguard the cross-institutional generalizability of the prognostic model in ESCC.

**Critical relevance statement:**

The exploration of repeatable RFs in different diseases and different types of imaging is conducive to promoting the proper use of radiomics in clinical research.

**Key Points:**

- The repeatability of RFs impacts the generalizability of the radiomic model.
- The high-repeatable RFs safeguard the cross-institutional generalizability of the model.
- Smaller bin width helps improve the repeatability of RFs.

**Graphical Abstract:**

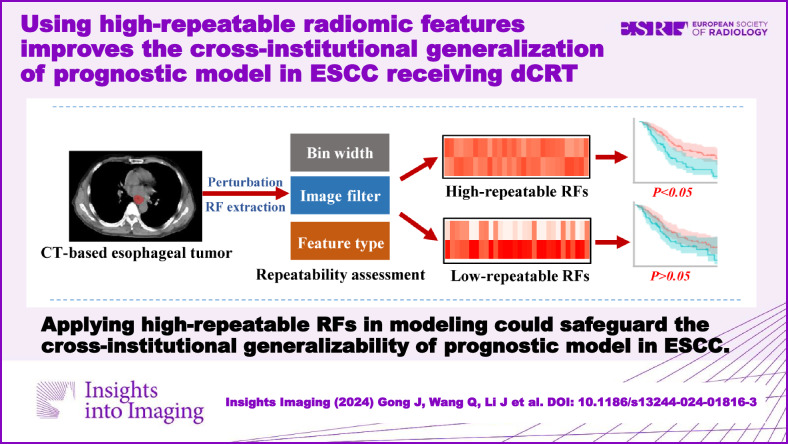

## Introduction

Contrast-enhanced computed tomography (CECT) performed routinely in clinical practice is widely used for radiomic modeling in esophageal cancer (EC) patients receiving definitive (chemo)radiotherapy (dCRT) [1–5]. It is worth noting that the stability and generalization of the radiomic model are the premises of its clinical translation, which might be directly affected by the repeatability of the radiomic feature (RF) [6].

A study of patients with head and neck cancer demonstrated that using robust CT RFs to establish the radiomic model significantly improves the robustness and generalizability of survival prognosis [7]. Another study provided further evidence that the radiomic model constructed by high-repeatable RFs from magnetic resonance imaging (MRI) had improved cross-institutional generalizability for disease-free survival in nasopharyngeal carcinoma [6]. It is necessary to carry out further investigations to verify whether this conclusion can be applied to other imaging modalities and cancer types. However, there is a lack of research on the repeatability of RF extracted from CECT in EC and its impact on cross-institutional generalization of the radiomic model.

The spatial variation of the tumor, caused by the uncertainties of scanning position and the inconsistencies of the tumor delineation, may affect the repeatability of RF [8–10]. While test-retest imaging, which involves repeating scans on the same patient, is recommended for assessing feature repeatability [11], it is not practical in the clinical setting for EC patients receiving dCRT. To address this limitation, a novel perturbation-based evaluation method for RF repeatability was proposed, which demonstrated similar patterns of feature repeatability to test-retest imaging [8]. Specifically, the image and mask are perturbed by performing an affine transformation that rotates them to simulate the effects of slight variations in patient posture or scanning angles. Additionally, the mask is perturbed through contour randomization by randomly selecting super voxels based on their overlap with the original mask, mimicking the boundary changes in the region of interest (ROI) due to imprecision in the segmentation process. This perturbation-based evaluation was effectively applied in non-small-cell lung cancer, nasopharyngeal carcinoma, and head-and-neck squamous cell carcinoma [6, 8]. Simulating uncertainties in the spatial position of patient scans using random rotation and inconsistencies in the delineation of regions of interest via contour randomization might be an effective perturbation-based method to evaluate RF repeatability in EC.

This study aims to investigate the repeatability of CECT-based RF via perturbation and its impact on the cross-institutional generalizability of the prognostic model for predicting local recurrence-free survival (LRFS) and overall survival (OS) in EC receiving dCRT. This study endeavors to demonstrate in EC that highly repeatable RFs contribute to enhancing the reliability and applicability of radiomic models, which might promote the appropriate use of radiomics in clinical research of EC, ultimately aiding in the advancement of personalized therapeutic approaches and strengthening clinical decision-making.

## Methods

### Patients

This study was approved by the Xijing Hospital Ethics Committee (KY20222145-C-1). The requirement for informed consent was waived because of a retrospective study. The major pathological type of EC is esophageal squamous cell carcinoma (ESCC), accounting for about 90% [12]. We enrolled ESCC patients who received dCRT or radiotherapy between February 2009 and June 2020 from Xijing Hospital and Sichuan Cancer Hospital, and collected their CECT images before radiotherapy. The inclusion/exclusion criteria are shown in Supplementary A1. The procedure of treatment and follow-up for LRFS and OS are described in Supplementary A2. Seven hundred ninety-two patients from Xijing Hospital and 120 patients from Sichuan Cancer Hospital were included in the training set and external validation set, respectively.

### Imaging acquisition, preprocessing, and tumor segmentation

**Fig. 1:**
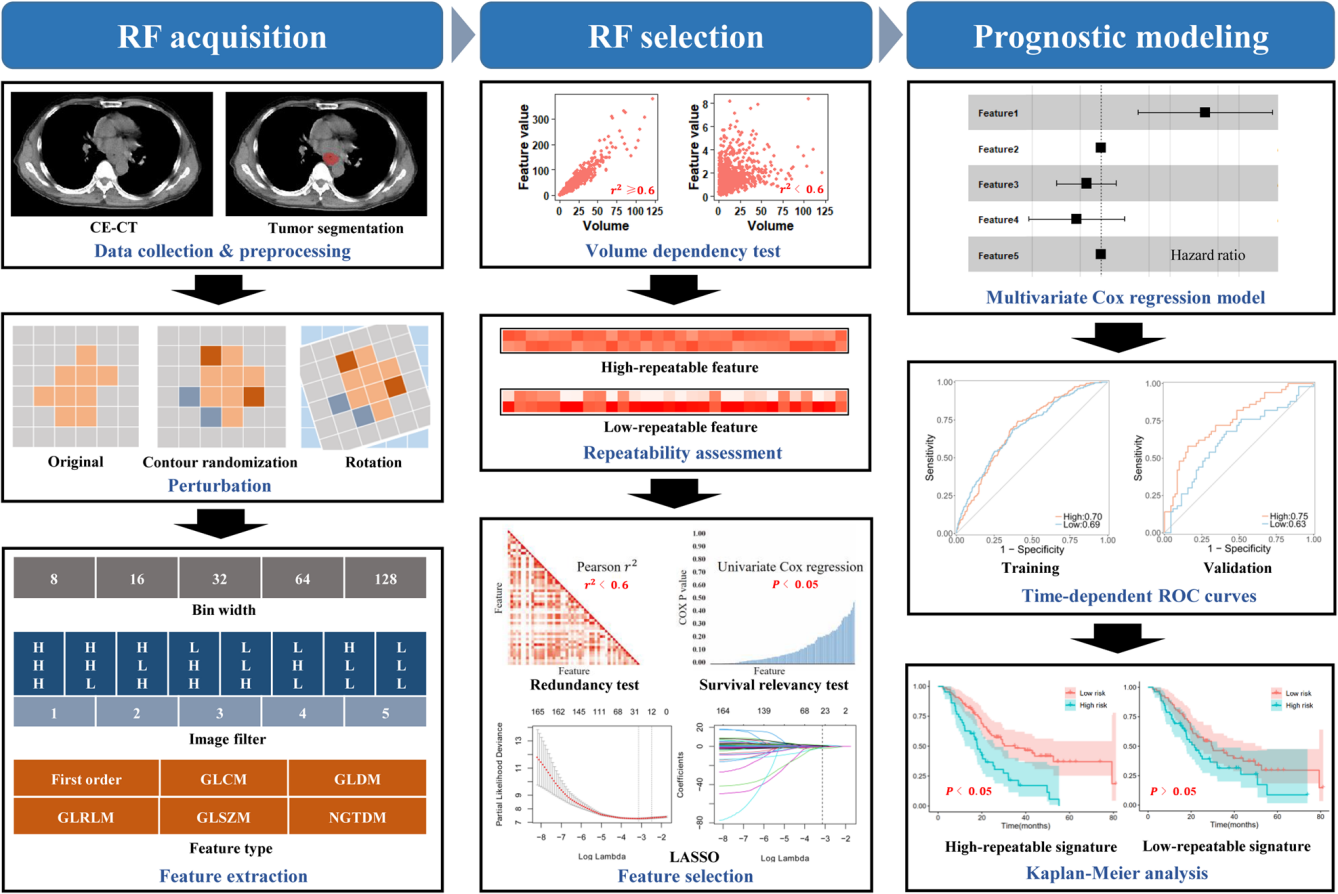
The workflow of the study. The images were preprocessed by the unified mediastinal window and resampled by 1 × 1 × 1 mm^3^, and the tumor was manually segmented. The spatial variation simulated by spatial rotation and contour randomization was implemented to generate image and ROI with perturbation. The 6510 RFs were extracted from the tumor and perturbed tumor separately to evaluate RF repeatability using ICC. The volume-independent features were equally grouped into high-repeatable and low-repeatable RF groups by the median ICC, which were further analyzed separately by multi-step feature selection and multivariate Cox proportional hazards regression model construction for predicting LRFS or OS. The *C*-index, time-dependent ROC, and Kaplan–Meier analysis were used to evaluate the prognostic performance of the model

The workflow is depicted in Fig. 1. The ESCC patients underwent a standard chest CECT scanning with scanners from the same manufacturer (Philips Healthcare, Cleveland OH, USA). The details of imaging acquisition and preprocessing parameters are shown in Tables S1 and S2. The gross tumor volume (GTV) was delineated as the region of interest (ROI) by one radiologist with five years of clinical diagnosing experience via the ITK-SNAP software (https://www.itksnap.org) and was corrected by two radiologists with 10 years of experience.

### Perturbation, RF extraction, and RF repeatability assessment

The spatial variation of the tumor caused by the differences in scanning position and the inconsistencies of the tumor delineation was simulated by spatial rotation and contour randomization, which were implemented with the parameters listed in Table S2. In total, 6510 RFs were extracted from the original and the perturbed images separately using PyRadiomics (https://pypi.org/project/pyradiomics/), which followed the guidelines proposed by the Image Biomarker Standardization Initiative [13]. Specifically, 18 first-order and 75 textural features were calculated from the original, 5 Laplacian-of-Gaussian (LoG)-filtered and 8 wavelet-filtered images, which were discretized by fixed bin width values of 8, 16, 32, 64, and 128 before feature extraction. Details of feature extraction are described in Supplementary A3. The intraclass correlation coefficient (ICC) is a statistical measure used to assess the reliability or consistency of quantitative measurements made by different raters or under different conditions, which is used to qualify RF repeatability [14, 15]. The one-way, random, absolute-agreement ICC of each RF was calculated by using values of this feature extracted from original and disturbed images of the training set. The value of ICC ranges from 0 to 1. A high ICC value indicates that the RF is robust and repeatable. The larger the value, the higher the repeatability.

### RF grouping and selection

The procedure of RF grouping and selection is also shown in Fig. 1. Primary tumor volume has been recognized as a reliable prognostic factor. In order to minimize the potential analytical bias caused by features strongly correlated with volume, we first conducted the volume dependency test with the square of the Pearson correlation coefficient (*r*^2^) greater than or equal to 0.6 as the criterion to remove RFs that were highly correlated with the mesh volume of the tumor. The volume-independent features were then equally grouped into high-repeatable and low-repeatable RF groups with the median ICC as the cutoff value, which were used to evaluate the impact of RF repeatability on the cross-institutional generalizability of the prognostic model. These two groups of features were analyzed separately in subsequent feature selection and prognostic model construction for predicting LRFS and OS. To simplify the model and reduce the possibility of overfitting, three steps including the redundancy test, survival relevancy test, and the least absolute shrinkage and selection operator (LASSO) Cox regression were used for feature selection, which is described in Supplementary A4.

### Performance comparison of radiomic models based on high- and low-repeatable RF groups

For LRFS and OS, feature selection and prognostic model construction were performed in both high- and low-repeatable groups, respectively. The features selected by LASSO were used to construct a prognostic model by multivariate Cox proportional hazards regression based on the training set, which was further validated in the validation set. The *C*-index was used to evaluate the performance of the model in the training set and validation set, and the area under the receiver operator characteristic curve (ROC) (AUC) was used to evaluate the classification ability at different time points including 1-year, 2-year, 3-year, and 5-year. Moreover, the prediction result of the model was used as the prognostic signature, and the median value of the training set was chosen as the threshold to stratify patients in both the training and validation sets into high-risk group and low-risk group, respectively. Kaplan–Meier analysis and log-rank test were used to evaluate the risk stratification ability.

### Performance comparison of radiomic models based on different thresholds of ICC

The bin width with the maximum average ICC might be suggested when extracting RFs from CECT in ESCC to obtain more repeatable features for construction of the radiomic model. Moreover, the choice of ICC threshold affects both model generalization and prognostic performance. Choosing too low of a threshold may fail to eliminate unstable features, leading to poorer model generalizability. Conversely, setting the threshold too high may exclude too many potentially informative prognostic features, resulting in a decrease in model performance. Therefore, we evaluated the performance and generalizability of models constructed at different ICC thresholds ranging from 0 to 1 in steps of 0.05, to select a reasonable threshold for constructing the final radiomic model.

### Prognostic performance of the clinical, radiomic, and fusion models

Univariate Cox regression analysis was performed on the training set to screen clinical factors related to survival, which served as inputs to multivariate Cox regression analysis to construct the clinical model. The performance of the clinical model, the final radiomic model, and the fusion model combining the clinical factors and the radiomic signature calculated by the radiomic model were measured quantitatively using the *C*-index in training and validation sets.

## Results

### Patient characteristics

The clinical characteristics of the training set from Xijing Hospital and the external validation set from Sichuan Cancer Hospital were listed in Table 1, and the median follow-up time was 75.0 and 46.7 months, respectively. The survival curves of LRFS and OS in training and validation sets are shown in Fig. S1.

**Table 1:** Characteristics of patients in the training and validation sets

Characteristics	Training (792)	Validation (120)	*p*
Age, (years)			< 0.05
< 70	492 (62.12)	101 (84.17)	
≥ 70	300 (37.88)	19 (15.83)	
Gender			0.77
Male	587 (64.02)	91 (75.83)	
Female	205 (25.88)	29 (24.17)	
PS			< 0.05
0–1	507 (64.02)	50 (41.67)	
2–3	285 (35.98)	70 (58.33)	
Location			< 0.05
Cervical/upper	168 (21.21)	58 (48.33)	
Middle	298 (36.63)	46 (38.33)	
Lower	326 (41.16)	16 (12.33)	
Tumor length, (cm)			0.15
< 6	470 (59.34)	80 (66.67)	
≥ 6	322 (40.66)	40 (33.33)	
*T*			0.06
1–3	554 (69.95)	73 (60.83)	
4	238 (30.05)	47 (39.17)	
*N*			< 0.05
0–1	642 (81.06)	53 (44.17)	
2–3	150 (18.94)	67 (55.83)	
PGTV dose, (Gy)			< 0.05
< 60	409 (51.64)	24 (20)	
≥ 60	383 (48.36)	96 (80)	
Concurrent chemotherapy			< 0.05
Without	160 (20.20)	10 (8.33)	
With	632 (79.80)	110 (91.67)	

The data is presented as numbers and percentages [*n* (%)]. The *p* values were calculated by the chi-square test

### Results of RF repeatability evaluation

**Fig. 2:**
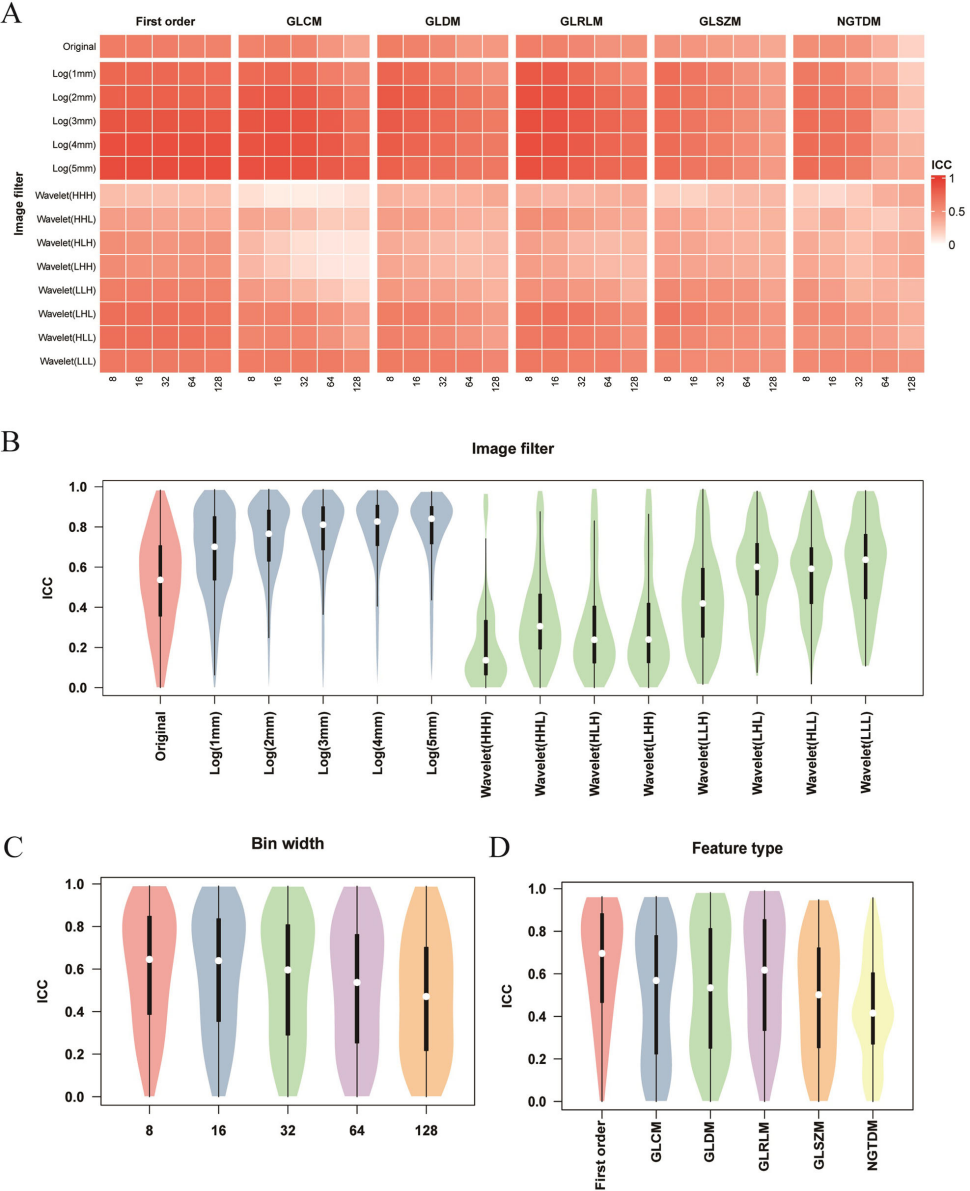
The distribution of ICC of RFs in different subgroups. **A** The mean ICC of RFs in different subgroups. The ICC of RFs extracted from different image filters (**B**), bin widths (**C**), and feature types (**D**)

Six thousand five hundred ten RFs were calculated from the original and the perturbed images separately. The ICC value of each RF pair was evaluated and the mean ICC of RFs from different feature types, different bin widths and different filters was assessed (Fig. 2). The first-order features from images processed by LoG filtering and smaller bin width were more repeatable, while texture features from images processed by wavelet filtering and larger bin width showed lower repeatability (Fig. 2A). In particular, RF from LoG filters had a good repeatability with the median ICC: 0.70–0.84, which increased with larger sigma (Fig. 2B). For wavelet filters, low-pass filter (LLL) had a higher repeatability with the median ICC of 0.64, and high-pass filter (HHH) had a lowest repeatability with the median ICC of 0.14. The repeatability of the original image was moderate with the median ICC of 0.54. For different bin widths, the features with parameters 8 had the highest repeatability with the median ICC of 0.65, which decreased with the increase of bin width (median ICC: 0.65–0.47) (Fig. 2C). For different feature types, first-order statistical features were more repeatable (median ICC: 0.70) than texture features (median ICC: 0.42–0.62), while NGTDM texture features had the lowest repeatability (median ICC: 0.42) (Fig. 2D).

### Results of high- and low-repeatable RF selection

The relationship between ICC and volume correlation *r*^2^ of all RFs is shown in Fig. S2. The removed volume-related 540 RFs had higher ICC values and stronger mean prognosis correlation than the retained 5970 RFs (median ICC: 0.89 vs 0.54; median − log_2_*p*: 28.13 vs 4.64). Then, the median ICC of 0.535 was used to divide the remaining 5970 RFs into a high-repeatable group (2985 RFs) and a low-repeatable group (2985 RFs).

The correlation of each retained RF with the rest of the features and its relationship to LRFS and OS was shown in Figs. S3 and S4, separately. One hundred and eighty-two high-repeatable RFs (182/2985) and 435 low-repeatable RFs (435/2985) passed the redundancy test, respectively. Then, 96 of high-repeatable RFs (96/182) and 169 of low-repeatable RFs (169/435) passed the LRFS correlation test (Fig. S5), while 99 of high-repeatable RFs (99/182) and 176 of low-repeatable RFs (176/435) passed the OS correlation test (Fig. S6). Based on LASSO, 26 high-repeatable and 25 low-repeatable RFs were separately selected for predicting LRFS, and 16 high-repeatable and 14 low-repeatable RFs were separately selected for predicting OS (Fig. S7). These four groups of RFs are detailed in Table S3–S6, and were used to construct multivariate Cox models for predicting LRFS and OS.

### Prognostic performance of models based on high- and low-repeatable RFs

**Fig. 3:**
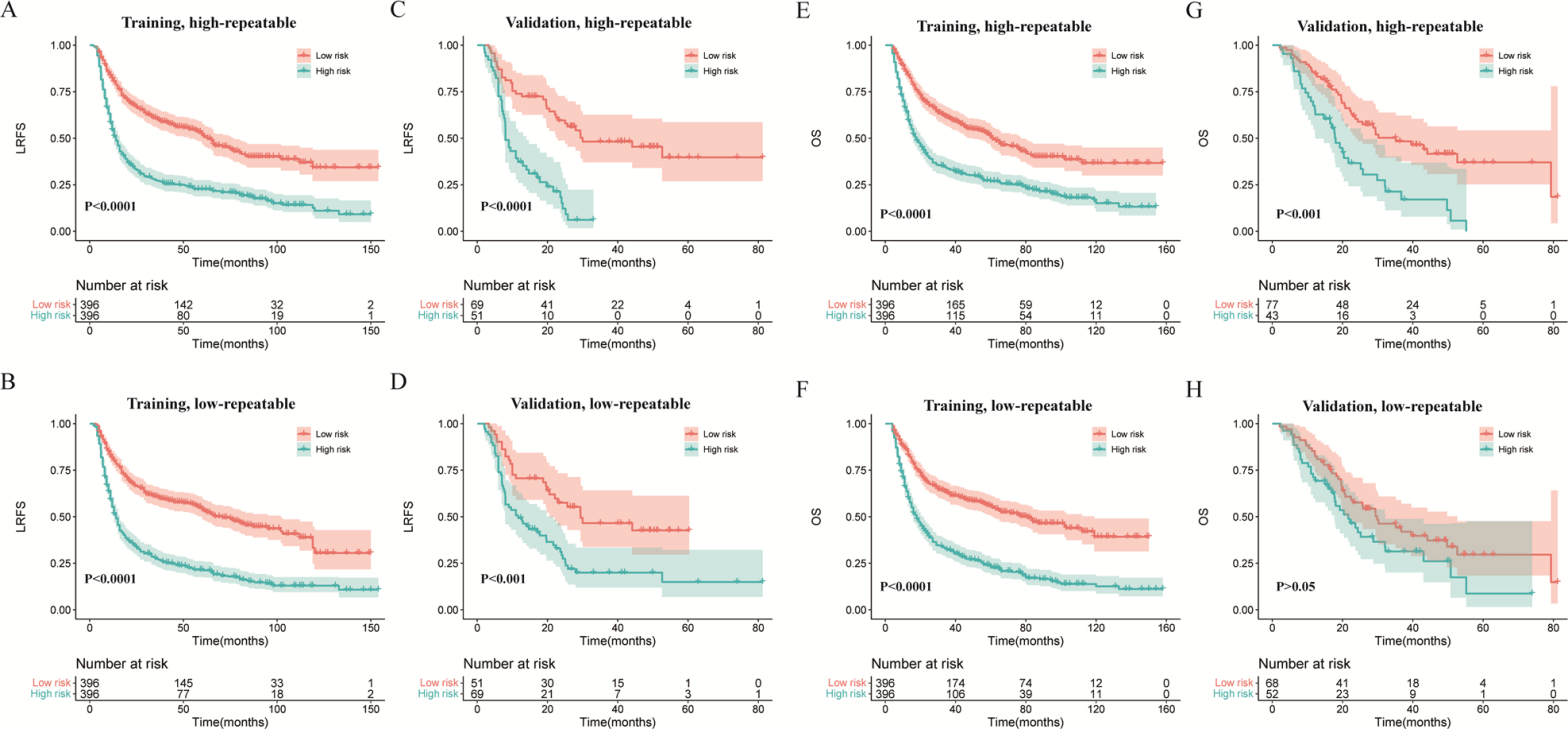
The Kaplan–Meier survival analyses of different LRFS and OS risk groups for high- and low-repeatable groups in training and validation sets. LRFS curves for high-repeatable groups in the training set (**A**), low-repeatable groups in the training set (**B**), high-repeatable groups in the validation set (**C**), and low-repeatable groups in the validation set (**D**). OS curves for high-repeatable groups in the training set (**E**), low-repeatable groups in the training set (**F**), high-repeatable groups in the validation set (**G**), low-repeatable groups in the validation set (**H**)

The performance of the multivariate Cox model based on high- and low-repeatable RFs remained stable in the training set, and *C*-index was similar (*C*-index for LRFS: 0.65 vs 0.67, *p* = 0.958; OS: 0.64 vs 0.65, *p* = 0.651) (Table 2). The signatures from the two models had a similar ability to screen patients with a high risk of local recurrence or death (hazard ratio (HR) for LRFS: 2.36 vs 2.51; OS: 2.1 vs 2.5), as shown in Fig. 3A, B, E, and F. The time-dependent AUC values were also similar between the high- and low-repeatable groups in the training set (Table 2), and the time-dependent ROCs were shown in Fig. S8A, C. However, in the external validation dataset, the model based on the high-repeatable group maintained similar performance to the training set, while the performance of the low-repeatable group was reduced. The *C*-index of the model developed by the high-repeatable group was significantly higher than that of the model developed by the low-repeatable group (*C*-index for LRFS: 0.67 vs 0.61, *p* = 0.013; OS: 0.63 vs 0.56, *p* = 0.013) (Table 2). Moreover, the signature from the low-repeatable RF model had a weaker ability to screen patients with high risk of local recurrence than that from the high-repeatable RF model in the external validation set (HR: 2.2 vs 3.6), as shown in Fig. 3C, D. The signature from the low-repeatable RF model had a weaker ability to screen patients with high risk of death than that from the high-repeatable RF model in the external validation set (HR: 1.5 vs 2.4), as shown in Fig. 3G, H. The time-dependent AUC of the model based on the low-repeatable group was also lower than that based on the high-repeatable group in the validation set, as shown in Table 2 and Fig. S8B, D.

**Table 2:** Prognostic performance of the high-repeatable features-based Cox model (high), the low-repeatable features-based Cox model (low) in training and validation sets

Endpoint	Metric	Training			Validation		
		High	Low	*p*	High	Low	*p*
LRFS	*C*-index	0.65 (0.62–0.67)	0.67 (0.64–0.69)	0.958	0.67 (0.61–0.73)	0.61 (0.55–0.67)	0.013
	1-y AUC	0.70 (0.66–0.73)	0.69 (0.65–0.73)	0.917	0.75 (0.66–0.84)	0.63 (0.53–0.73)	0.004
	2-y AUC	0.70 (0.66–0.74)	0.71 (0.67–0.75)	0.452	0.79 (0.70–0.87)	0.70 (0.60–0.80)	0.024
	3-y AUC	0.68 (0.64–0.72)	0.70 (0.67–0.75)	0.130	0.79 (0.70–0.88)	0.71 (0.61–0.82)	0.123
	5-y AUC	0.63 (0.58–0.68)	0.69 (0.64–0.74)	0.005	0.72 (0.56–0.88)	0.58 (0.45–0.70)	0.145
OS	*C*-index	0.64 (0.62–0.67)	0.65 (0.62–0.67)	0.651	0.63 (0.57–0.69)	0.56 (0.50–0.63)	0.013
	1-y AUC	0.69 (0.65–0.73)	0.69 (0.65–0.73)	0.938	0.66 (0.55–0.77)	0.58 (0.46–0.70)	0.095
	2-y AUC	0.68 (0.64–0.72)	0.68 (0.64–0.72)	0.899	0.68 (0.57–0.78)	0.58 (0.48–0.69)	0.045
	3-y AUC	0.66 (0.62–0.70)	0.68 (0.64–0.72)	0.546	0.69 (0.58–0.80)	0.58 (0.46–0.71)	0.072
	5-y AUC	0.61 (0.56–0.66)	0.66 (0.61–0.71)	0.007	0.73 (0.59–0.99)	0.59 (0.35–0.84)	0.300

*p* values were calculated by the Student *t*-test for the comparison of the *C*-index of high and that of low or the DeLong’s test for the comparison of the AUC of high and that of low

### Prognostic performance of the clinical, radiomic, and fusion models

**Fig. 4:**
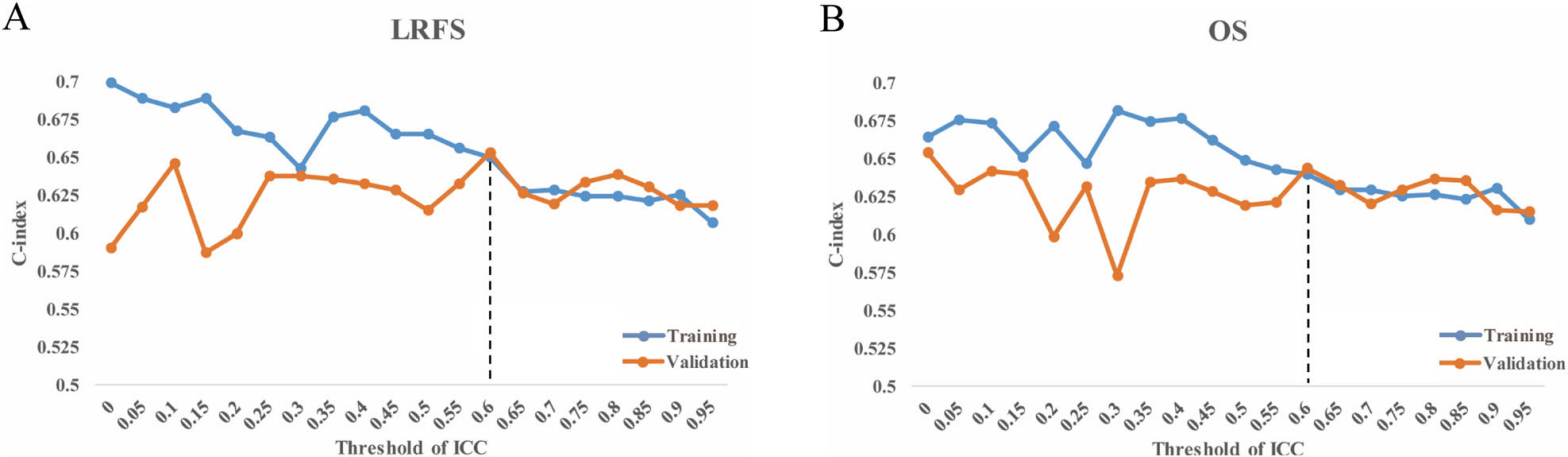
The *C*-index of models based on different thresholds of ICC for predicting LRFS (**A**) or OS (**B**)

Whether LRFS or OS, clinical factors including age, tumor location, tumor length, T stage, and concurrent chemotherapy were selected by univariate analysis (*p* < 0.05) and were used to construct the clinical models (Table S7). The RFs extracted by bin width of 8 were used to evaluate the performance of models based on different thresholds of ICC for predicting LRFS or OS (Fig. 4A, B). The ICC threshold was set as 0.6 to guarantee model performance and generalization. The performance of the clinical, radiomic, and fusion models and the comparison were summarized in Table 3. The dynamic nomograms of the fusion models are available online at https://eclrfs.shinyapps.io/RF_repeatability_LRFS/ and https://eclrfs.shinyapps.io/RF_repeatability_OS/, which could interactively calculate the specific LRFS and OS probability of ESCC received dCRT, respectively.

**Table 3:** The *C*-index of the clinical model, the final radiomic model, and the fusion model in training and validation sets for predicting LRFS or OS

Endpoint	Model	Training		Validation	
		*C*-index	*p*	*C*-index	*p*
LRFS	Clinical model	0.62 (0.59–0.65)		0.60 (0.0.53–0.68)	
	Radiomic model	0.65 (0.62–0.67)	0.026	0.65 (0.59–0.72)	0.112
	Fusion model	0.67 (0.65–0.70)	< 0.001	0.65 (0.0.58–0.71)	0.072
OS	Clinical model	0.62 (0.59–0.65)		0.57 (0.50–0.64)	
	Radiomic model	0.64 (0.61–0.66)	0.137	0.64 (0.58–0.71)	0.024
	Fusion model	0.66 (0.64–0.69)	< 0.001	0.60 (0.53–0.66)	0.107

*p* values were calculated by the Student *t*-test to compare the *C*-index of radiomics-related models with that of the clinical model

## Discussion

In this study, we investigated the repeatability of CECT-based RF via spatial perturbation and demonstrated its impact on the cross-institutional generalizability of the prognostic model for predicting LRFS and OS in ESCC receiving dCRT. The first-order features from images processed by LoG filtering and smaller bin width were more repeatable, while texture features from images processed by wavelet filtering and larger bin width were less repeatable. The high- and low-repeatable RFs remained similar prognostic performance in the training set (*p* > 0.05), while the performance of the model based on the low-repeatable RF group was significantly lower than that based on the high-repeatable RF group in the external validation set (*p* < 0.05). The high-repeatable RFs for modeling could safeguard the cross-institutional generalizability of the prognostic model for wide-spreading clinical utilization in EC.

This study is the first to assess the impact of CECT-based RF repeatability via spatial perturbation on the cross-institutional generalization ability of prognostic models in EC. Having two repeat scans of the same patient is recommended to assess the repeatability of features [11]. However, test-retest imaging is not feasible in clinical practice for EC patients receiving dCRT because of tumor shrinkage or potential radiation damage. Moreover, manual segmentation of the same tumor multiple times is often used to select highly repeatable features but is time-consuming and laborious [9]. These objective factors may lead to the lack of investigations about the impact of RF repeatability in EC. However, the data perturbation simulation technique proposed by Zwanenburg et al [8] might be an effective solution and has been successfully applied to the studies of the RF repeatability in non-small cell lung cancer, nasopharyngeal carcinoma, and head and neck squamous cell carcinoma [6, 8]. We also evaluated the repeatability of RF in EC using the perturbation-based method.

The trend of RF repeatability of different feature types, filters, and bin widths in EC is consistent with previous studies [6]. The repeatability of first-order statistical features is higher than that of texture features. These features are not influenced by the spatial arrangement of the pixels, making them less sensitive to small variations in the image. In different filters, LoG is more repeatable than wavelet, and the repeatability improves with the increase of sigma [16]. This observation can be attributed to the smoothing effect of the LoG filter, which reduces noise and enhances edges, potentially leading to more stable features. Additionally, we noted that the repeatability of features improved as the sigma value increased. A higher sigma value corresponds to a larger scale of the filter, which can further reduce noise and enhance the robustness of the extracted features [17]. Among wavelet filters, high-pass filters do have lower repeatability because of their sensitivity to minute variations in the image [11, 18]. In addition, a larger width of the bin results in more gray values merging, which may result in a loss of texture information and lead to greater changes in texture features. Although different studies may use different methods to evaluate the RF repeatability for different diseases [7, 8, 16], the consistent trend reflects the generalization of RF repeatability. The RF may have similar repeatability in different tumors due to its definition, but validation in specific situations is still necessary for helping the conclusion generalization and clinical application.

This study provided direct evidence that CECT-based RF repeatability affects the cross-institutional generalization of the prognosis model in ESCC. For both OS and LRFS prediction, the prognostic performance of the high and low-repeatable RF groups was similar in the training set, while the external validation performance of the model from the low-repeatable RF group was significantly reduced. Previous studies have proposed the effect of enhanced magnetic resonance feature repeatability on the generalization performance of disease-free survival based on nasopharyngeal carcinoma [6]. In this study, contrast-enhanced CT was also used to verify different prognostic endpoints including LRFS and OS in EC, adding more evidence for the conclusion that feature repeatability might affect the generalization of the prognostic model. Providing further evidence for more diseases, more modalities and more endpoints is to be encouraged. The establishment of a pan-cancer feature list with high repeatability may be helpful for subsequent radiomics-related studies to build more stable models that are more suitable for clinical application.

In the high-repeatable group, features mainly from Firstorder and GLCM classes are more likely to capture stable and meaningful information about the underlying tissue structure and pathology, making them suitable for clinical application and model building. In contrast, the low-repeatable group includes more GLRLM, GLSZM, and NGTDM features, which capture more variable information about the size and distribution of homogeneous regions. Although these features may provide insights into the complexity of the tumor microenvironment, their low repeatability severely limits their feasibility for model building. In any case, the clinical utility of a diagnostic or prognostic tool should prioritize stability and reliability.

There are still some limitations. This study only included data from two centers for the analysis of RF repeatability and its impact on model generalizability in ESCC. Future research should involve larger samples, more centers, and diverse clinical scenarios to validate our findings and explore other factors affecting model generalizability. Although the perturbation algorithm is feasible and effective as a feature stability assessment for exploratory analysis, future prospective experiments are still needed to further validate our results in a real-world scenario. Moreover, many factors such as acquisition [19], reconstruction [20], or preprocessing [21, 22] also affect the feature repeatability [23]. It is necessary to further explore the influence of various processing steps to minimize the interference to the features. Finally, radiomics has the potential to predict the prognosis of EC. The fusion model with added radiomics was superior to the clinical model, but the external validation set might be limited by sample size or clinical differences in patient distribution, resulting in insignificance. So we developed online nomograms to encourage more centers to validate the results for improving the reliability of our models. Moreover, the performance of prognostic models based on radiomics is often limited in cancer research [2, 24–26], which might be attributed to a combination of factors, including data heterogeneity, limited sample size, feature selection, and stability issues, clinical complexity, external validation challenges, and technological limitations. Rigorous data collection, selection of repeatable features, model validation, and multicenter collaboration help improve the performance and clinical applicability of prognostic models. From the perspective of data information and technology, considering subregional radiomics, deep learning algorithms, multimodal images or other omics information might improve the performance of the model [2, 24–28].

## Conclusion

In conclusion, we provided evidence that highly repeatable RFs could safeguard the cross-institutional generalizability of prognostic models and improve the prognostic performance of clinical models in ESCC. We recommend conducting a systematic assessment of RF repeatability and incorporating only highly repeatable features into predictive models to enhance their generalizability and clinical applicability.

## Supplementary information

ELECTRONIC SUPPLEMENTARY MATERIAL

## Data Availability

Data are available from the corresponding author upon request.

## Abbreviations

AUC: Area under the receiver operator characteristic curve
CECT: Contrast-enhanced computed tomography
dCRT: Definitive chemoradiotherapy
EC: Esophageal cancer
ESCC: Esophageal squamous cell cancer
HR: Hazard ratio
ICC: Intraclass correlation coefficient
LASSO: Least absolute shrinkage and selection operator
LRFS: Local recurrence-free survival
MRI: Magnetic resonance imaging
OS: Overall survival
RF: Radiomic feature
ROC: Receiver operator characteristic curve

## References

[1] Hou Z, Ren W, Li S et al (2017) Radiomic analysis in contrast-enhanced CT: predict treatment response to chemoradiotherapy in esophageal carcinoma. Oncotarget 8:104444–104454. 10.18632/oncotarget.22304 29262652 10.18632/oncotarget.22304 PMC5732818

[2] Gong J, Zhang W, Huang W et al (2022) CT-based radiomics nomogram may predict local recurrence-free survival in esophageal cancer patients receiving definitive chemoradiation or radiotherapy: a multicenter study. Radiother Oncol 174:8–15. 10.1016/j.radonc.2022.06.010 35750106 10.1016/j.radonc.2022.06.010

[3] Cao B, Mi K, Dai W et al (2022) Prognostic and incremental value of computed tomography-based radiomics from tumor and nodal regions in esophageal squamous cell carcinoma. Chin J Cancer Res 34:71–82. 10.21147/j.issn.1000-9604.2022.02.02 35685995 10.21147/j.issn.1000-9604.2022.02.02 PMC9086572

[4] Gu L, Liu Y, Guo X et al (2021) Computed tomography-based radiomic analysis for prediction of treatment response to salvage chemoradiotherapy for locoregional lymph node recurrence after curative esophagectomy. J Appl Clin Med Phys 22:71–79. 10.1002/acm2.13434 10.1002/acm2.13434 PMC8598151 34614265

[5] Qiu Q, Duan J, Deng H et al (2020) Development and validation of a radiomics nomogram model for predicting postoperative recurrence in patients with esophageal squamous cell cancer who achieved pCR after neoadjuvant chemoradiotherapy followed by surgery. Front Oncol 10:1398. 10.3389/fonc.2020.01398 32850451 10.3389/fonc.2020.01398 PMC7431604

[6] Zhang J, Lam S-K, Teng X et al (2023) Radiomic feature repeatability and its impact on prognostic model generalizability: a multi-institutional study on nasopharyngeal carcinoma patients. Radiother Oncol 183:109578. 10.1016/j.radonc.2023.109578 36822357 10.1016/j.radonc.2023.109578

[7] Teng X, Zhang J, Ma Z et al (2022) Improving radiomic model reliability using robust features from perturbations for head-and-neck carcinoma. Front Oncol 12:974467. 10.3389/fonc.2022.974467 36313629 10.3389/fonc.2022.974467 PMC9614273

[8] Zwanenburg A, Leger S, Agolli L et al (2019) Assessing robustness of radiomic features by image perturbation. Sci Rep 9:614. 10.1038/s41598-018-36938-4 30679599 10.1038/s41598-018-36938-4 PMC6345842

[9] Leijenaar RTH, Carvalho S, Velazquez ER et al (2013) Stability of FDG-PET radiomics features: an integrated analysis of test–retest and inter-observer variability. Acta Oncol 52:1391–1397. 10.3109/0284186X.2013.812798 24047337 10.3109/0284186X.2013.812798 PMC4533992

[10] Desseroit M-C, Tixier F, Weber WA et al (2017) Reliability of PET/CT shape and heterogeneity features in functional and morphologic components of non-small cell lung cancer tumors: a repeatability analysis in a prospective multicenter cohort. J Nucl Med 58:406–411. 10.2967/jnumed.116.180919 27765856 10.2967/jnumed.116.180919 PMC5331937

[11] van Timmeren JE, Leijenaar RTH, van Elmpt W et al (2016) Test–retest data for radiomics feature stability analysis: generalizable or study-specific? Tomography 2:361–365. 10.18383/j.tom.2016.00208 30042967 10.18383/j.tom.2016.00208 PMC6037932

[12] Rustgi AK, El-Serag HB (2014) Esophageal carcinoma. N Engl J Med 371:2499–2509. 10.1056/NEJMra1314530 25539106 10.1056/NEJMra1314530

[13] Zwanenburg A, Vallières M, Abdalah MA et al (2020) The image biomarker standardization initiative: standardized quantitative radiomics for high-throughput image-based phenotyping. Radiology 295:328–338. 10.1148/radiol.2020191145 32154773 10.1148/radiol.2020191145 PMC7193906

[14] Shrout PE, Fleiss JL (1979) Intraclass correlations: uses in assessing rater reliability. Psychol Bull 86:420–428. 10.1037//0033-2909.86.2.420 18839484 10.1037//0033-2909.86.2.420

[15] Xue C, Yuan J, Lo GG et al (2021) Radiomics feature reliability assessed by intraclass correlation coefficient: a systematic review. Quant Imaging Med Surg 11:4431–4460. 10.21037/qims-21-86 34603997 10.21037/qims-21-86 PMC8408801

[16] Shiri I, Hajianfar G, Sohrabi A et al (2020) Repeatability of radiomic features in magnetic resonance imaging of glioblastoma: test–retest and image registration analyses. Med Phys 47:4265–4280. 10.1002/mp.14368 32615647 10.1002/mp.14368

[17] Barry N, Rowshanfarzad P, Francis RJ et al (2021) Repeatability of image features extracted from FET PET in application to post-surgical glioblastoma assessment. Phys Eng Sci Med 44:1131–1140. 10.1007/s13246-021-01049-4 34436751 10.1007/s13246-021-01049-4

[18] Tunali I, Hall LO, Napel S et al (2019) Stability and reproducibility of computed tomography radiomic features extracted from peritumoral regions of lung cancer lesions. Med Phys 46:5075–5085. 10.1002/mp.13808 31494946 10.1002/mp.13808 PMC6842054

[19] Ligero M, Jordi-Ollero O, Bernatowicz K et al (2021) Minimizing acquisition-related radiomics variability by image resampling and batch effect correction to allow for large-scale data analysis. Eur Radiol 31:1460–1470. 10.1007/s00330-020-07174-0 32909055 10.1007/s00330-020-07174-0 PMC7880962

[20] Larue RTHM, Van Timmeren JE, De Jong EEC et al (2017) Influence of gray level discretization on radiomic feature stability for different CT scanners, tube currents and slice thicknesses: a comprehensive phantom study. Acta Oncol 56:1544–1553. 10.1080/0284186X.2017.1351624 28885084 10.1080/0284186X.2017.1351624

[21] Wichtmann BD, Harder FN, Weiss K et al (2023) Influence of image processing on radiomic features from magnetic resonance imaging. Invest Radiol 58:199–208. 10.1097/RLI.0000000000000921 36070524 10.1097/RLI.0000000000000921

[22] Park S-H, Lim H, Bae BK et al (2021) Robustness of magnetic resonance radiomic features to pixel size resampling and interpolation in patients with cervical cancer. Cancer Imaging 21:19. 10.1186/s40644-021-00388-5 33531073 10.1186/s40644-021-00388-5 PMC7856733

[23] Zhao B (2021) Understanding sources of variation to improve the reproducibility of radiomics. Front Oncol 11:633176. 10.3389/fonc.2021.633176 33854969 10.3389/fonc.2021.633176 PMC8039446

[24] Xie C, Yang P, Zhang X et al (2019) Sub-region based radiomics analysis for survival prediction in oesophageal tumours treated by definitive concurrent chemoradiotherapy. EBioMedicine 44:289–297. 10.1016/j.ebiom.2019.05.023 31129097 10.1016/j.ebiom.2019.05.023 PMC6606893

[25] Forouzannezhad P, Maes D, Hippe DS et al (2022) Multitask learning radiomics on longitudinal imaging to predict survival outcomes following risk-adaptive chemoradiation for non-small cell lung cancer. Cancers 14:1228. 10.3390/cancers14051228 35267535 10.3390/cancers14051228 PMC8909466

[26] Li H-J, Liu L-Z, Huang Y et al (2022) Establishment and validation of a novel MRI radiomics feature-based prognostic model to predict distant metastasis in endemic nasopharyngeal carcinoma. Front Oncol 12:794975. 10.3389/fonc.2022.794975 35402262 10.3389/fonc.2022.794975 PMC8983880

[27] Beukinga RJ, Wang D, Karrenbeld A et al (2021) Addition of HER2 and CD44 to 18F-FDG PET-based clinico-radiomic models enhances prediction of neoadjuvant chemoradiotherapy response in esophageal cancer. Eur Radiol 31:3306–3314. 10.1007/s00330-020-07439-8 33151397 10.1007/s00330-020-07439-8 PMC8043921

[28] Jin X, Zheng X, Chen D et al (2019) Prediction of response after chemoradiation for esophageal cancer using a combination of dosimetry and CT radiomics. Eur Radiol 29:6080–6088. 10.1007/s00330-019-06193-w 31028447 10.1007/s00330-019-06193-w

