# Supplementary material for: Using high-repeatable radiomic features improves the cross-institutional generalization of prognostic model in esophageal squamous cell cancer receiving definitive chemoradiotherapy
Source: Insights Imaging. 2024 Oct 7;15:239. doi: 10.1186/s13244-024-01816-3 (PMC11458848; doi:10.1186/s13244-024-01816-3)
Supplement: Supplementary file 1 — ELECTRONIC SUPPLEMENTARY MATERIAL [file 13244_2024_1816_MOESM1_ESM.pdf]

Using high-repeatable radiomic features improves the cross-institutional generalization of prognostic model in esophageal squamous cell cancer receiving definitive chemoradiotherapy

ELECTRONIC SUPPLEMENTAL MATERIAL

Supplementary A1. The inclusion/exclusion criteria of participants.

Inclusion criteria: (1) pathological biopsy was diagnosed as ESCC; (2) CECT within 1 week before treatment; (3) definitive radiation dose>50Gy. Exclusion criteria: (1) patients received esophagectomy and preoperative or postoperative adjuvant radiotherapy; (2) patients without follow-up information or with incomplete clinical information; (3) low-quality CT imaging.

Supplementary A2. The procedure of treatment and follow-up.

The majority of ESCC patients received definitive (chemo)radiotherapy and for patients with advanced ages, poor performance status or patient request, radiotherapy alone was delivered to these patients. The definition and dose prescription of radiotherapy target volumes and dose constraints to the organs at risk followed the protocols reported by the study [1]. A total prescribed dose of 50-70 Gy to the planning target volume (PTV) in 25 to 35 fractions was delivered to the patients. For chemotherapy regimen, more than half of the patients received Fluorouracil/capecitabine and cisplatin (PF) chemotherapy and part of patients received capecitabine or S1. The choice of

chemotherapy was based on multidisciplinary team decision and preference of patients. After radiotherapy, follow-up evaluations were performed once a month in the first year, every 3 months for the first 2 years, every 6 months for 3-5 years, and once a year thereafter. Each evaluation included the physical examination, the blood test, the barium esophagram, the CT scan of the neck and chest, and the abdominal ultrasound. The endoscopy and biopsy were performed once local recurrence was suspected. Patients were followed up for more than 2 years after treatment. LRFS was defined as the time from the first date of therapy until the date of local recurrence or death, and OS was defined as the time from the first date of therapy until the date of death.

Supplementary A3. The details of feature extraction.

The 6,510 RFs and 6,510 RFs under perturbation were extracted from the tumor from unperturbed tumor and perturbed tumor separately using PyRadiomics. Specifically, 18 first-order intensity statistics features and 75 textural features consisting of 24 gray level co-occurrence texture matrices (GLCM) features, 14 gray level dependence matrix (GLDM) features, 16 gray level run-length texture matrices (GLRLM) features, 16 gray level size zone matrix (GLSZM) features and 5 neighborhood gray tone difference matrix (NGTDM) features were calculated from the original, 5 Laplacian-of-Gaussian (LoG) filtered and 8 wavelet-filtered images, which was discretized by fixed bin width values of 8, 16, 32, 64, and 128 before feature extraction. The parameters of feature extraction were also listed in **Table S2**.

Supplementary A4. The details of feature selection.

The three steps of feature selection including the redundancy test, survival relevancy test and the least absolute shrinkage and selection operator (LASSO) Cox regression. First, the redundancy test was used to reduce the co-linearity of the features. For each highly correlated feature pair with r^2 greater than or equal to 0.6, the one that has a larger mean r^2 with the rest of the features was removed. Then, the survival relevancy test was performed by univariate Cox regression with $P<0.05$. Finally, the LASSO Cox regression model was applied to select the most predictive features. The parameter λ, which controls the strength of regularization, was optimized using ten-fold cross-validation repeated 100 times via minimum criteria. Features with non-zero coefficients were selected for final prognostic model construction and validation.

References:

[1] Zhao L, Zhou Y, Pan H, Yin Y, Chai G, Mu Y, et al. Radiotherapy Alone or Concurrent Chemoradiation for Esophageal Squamous Cell Carcinoma in Elderly Patients. J Cancer. 2017;8:3242-50.

Supplemental Figures

Figure S1. The survival curves of LRFS and OS in training and validation sets. The median LRFS and OS of the training set were 25.0 months (95% CI: 21.0–30.0 months) and 30.0 months (95% CI: 25.0–38.0 months). The median LRFS and OS of the validation set were 19.7 months (95% CI: 12.7–25.0 months) and 25.1 months (95% CI: 20.5–35.1 months).

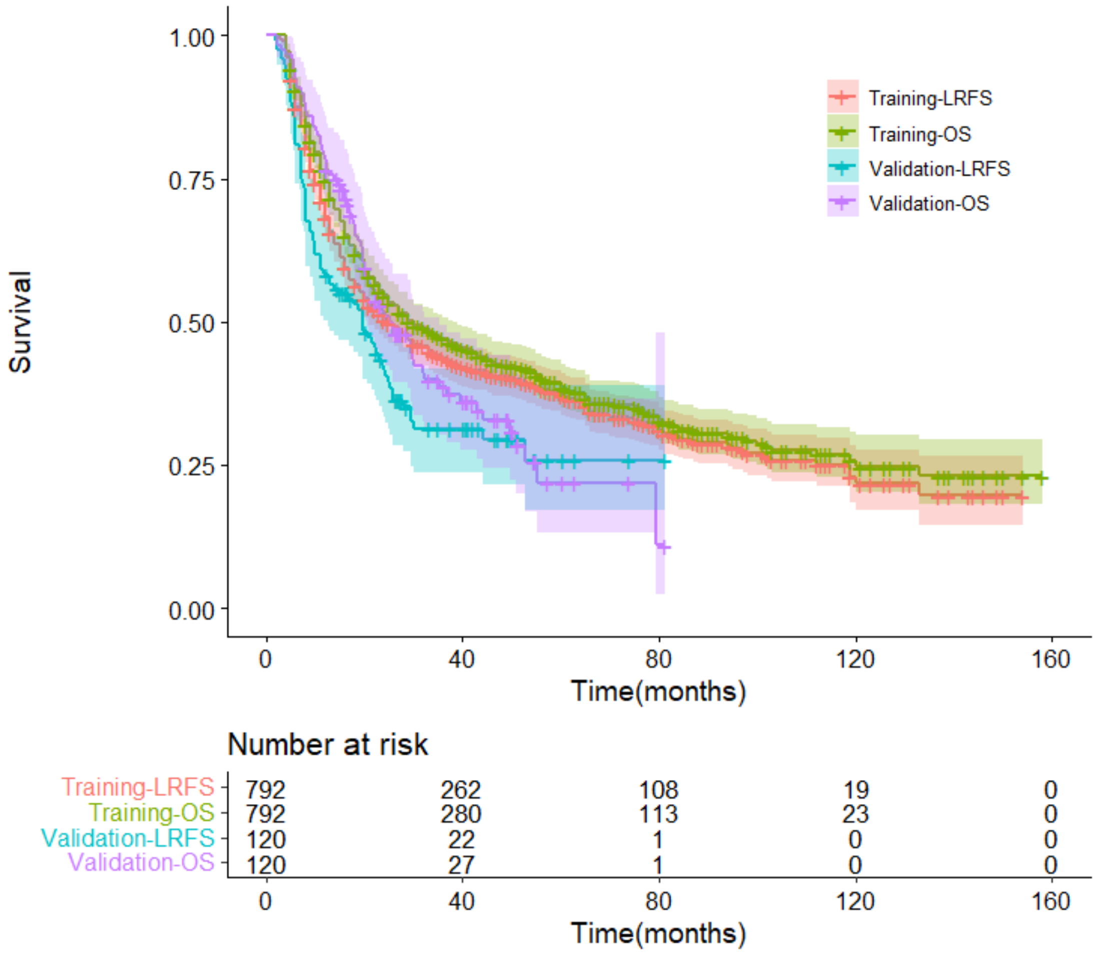

Figure S2. The relationship between repeatability and volume correlation of all RFs. Each point represents a radiomic feature whose horizontal coordinate is the square of the Pearson correlation coefficient (r^2) from the volume dependency test, and whose vertical coordinate is ICC calculated by features from unperturbed tumor and perturbed tumor. The r^2 greater than or equal to 0.6 was used as the criterion to remove RFs that were highly correlated with mesh volume of tumor. The blue dots are volume-independent features and the pink dots are volume-correlated features.

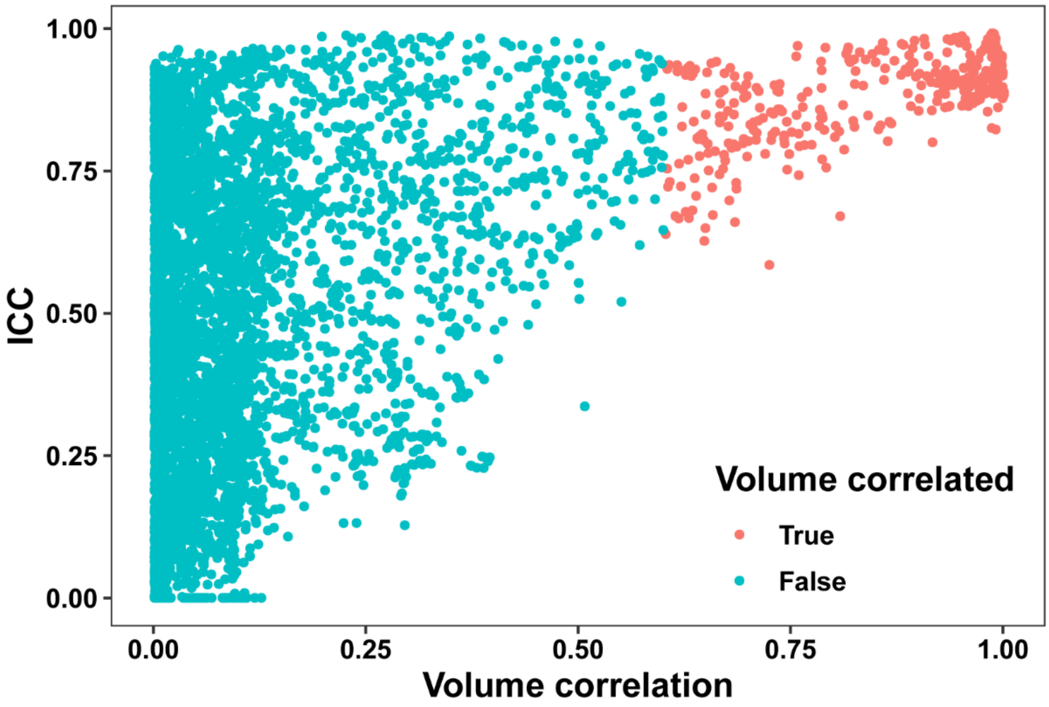

Figure S3. The distribution of volume-independent RFs before redundancy and LRFS relevancy test. Each point represents a radiomic feature whose horizontal coordinate is the $-\log_2 P$ evaluated by the LRFS relevancy test using univariate Cox regression, and whose vertical coordinate is the mean of the square of Pearson correlation coefficient(r^2) for each feature with the rest of features. The blue dots are low-repeatable features and the pink dots are high-repeatable features.

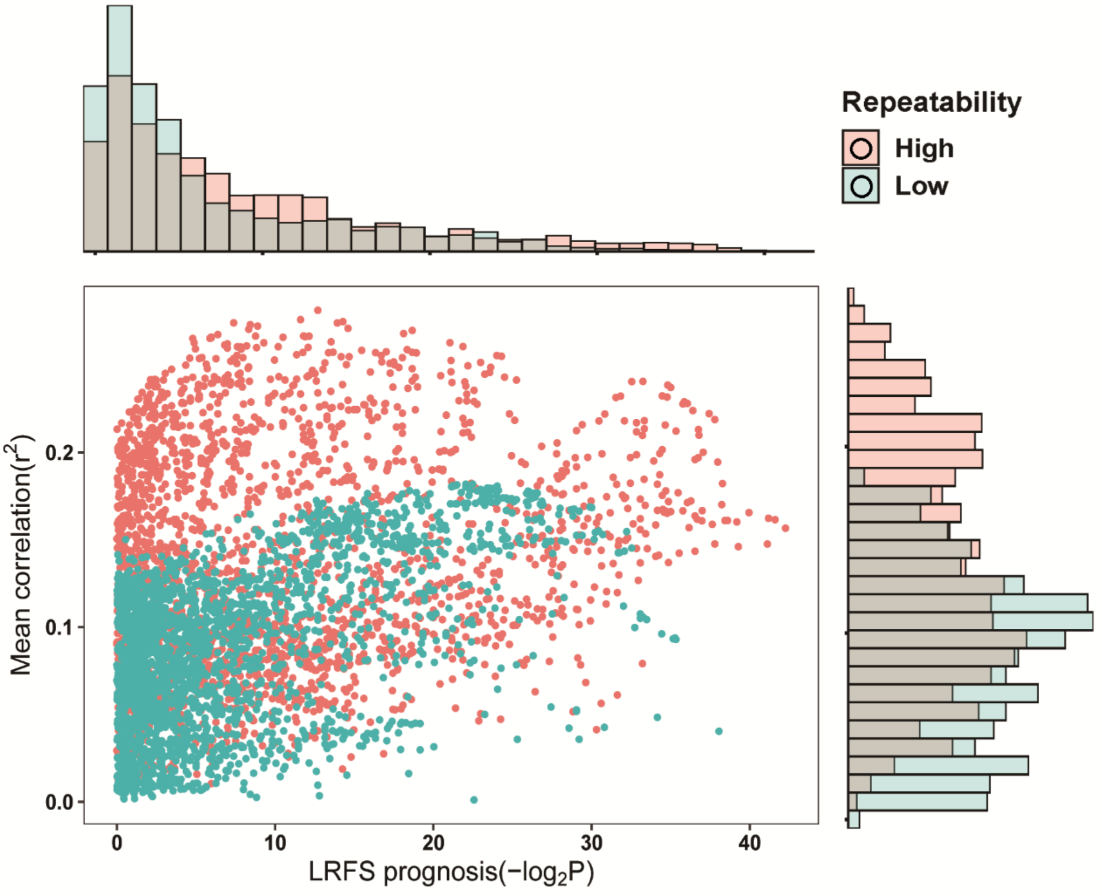

Figure S4. The distribution of volume-independent RFs before redundancy and OS relevancy test. Each point represents a radiomic feature whose horizontal coordinate is the $-\log_2 P$ evaluated by the OS relevancy test using univariate Cox regression, and whose vertical coordinate is the mean of the square of Pearson correlation coefficient(r^2) for each feature with the rest of features. The blue dots are low-repeatable features and the pink dots are high-repeatable features.

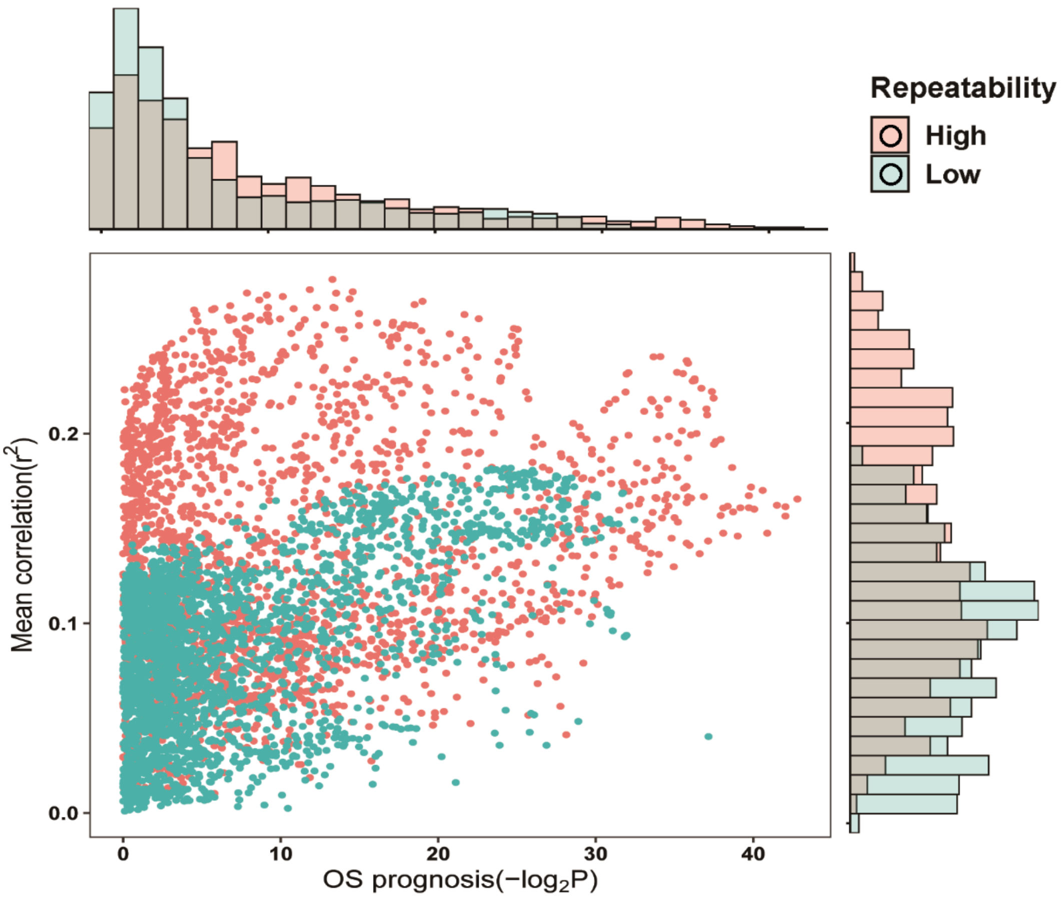

Figure S5. The distribution of RFs after redundancy and LRFS relevancy test. For each highly correlated feature pair with r^2 greater than or equal to 0.6, the one that has a larger mean r^2 was removed. Each point represents a RF whose horizontal coordinate is the $-\log_2 P$ evaluated by the LRFS relevancy test using univariate Cox regression, and whose vertical coordinate is the mean of the square of Pearson correlation coefficient(r^2) for each feature with the rest of features. The blue dots are low-repeatable features and the pink dots are high-repeatable features. The triangular dots were the feature selected by LRFS relevancy test with $P<0.05$. The round dots failed the redundancy test and should be removed.

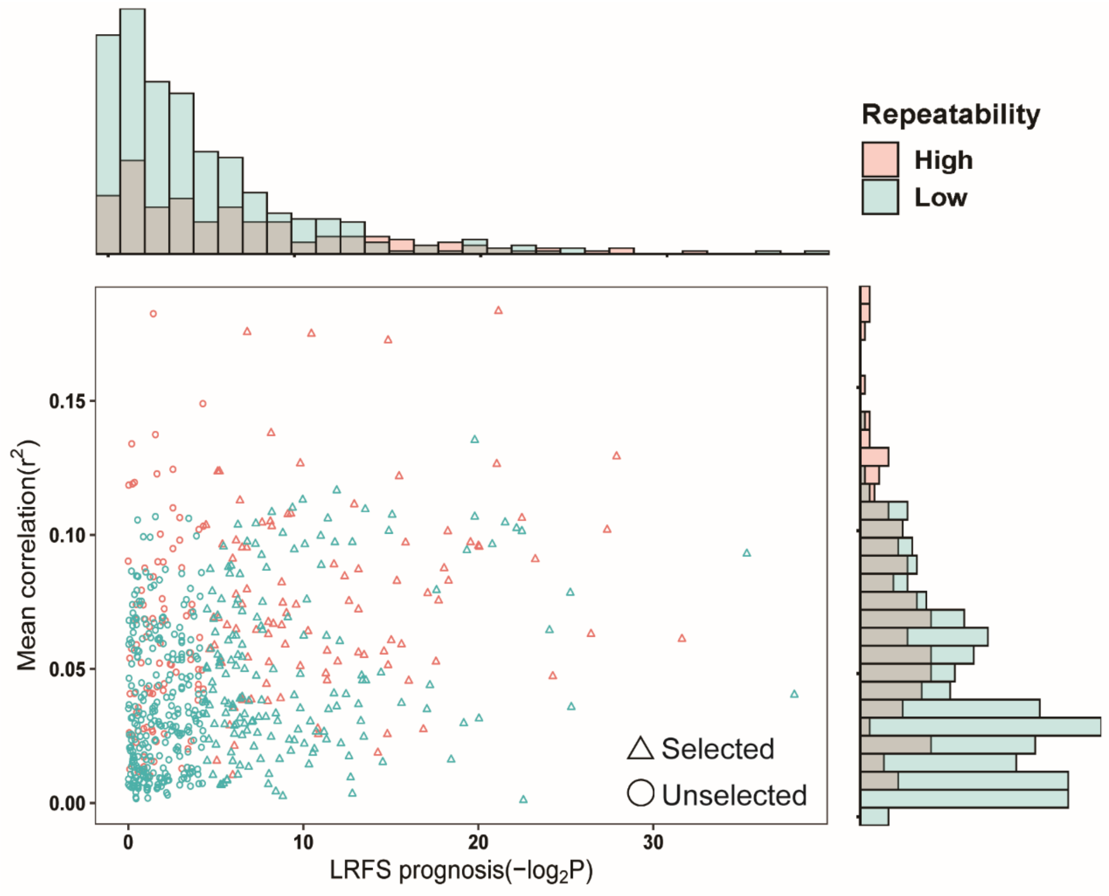

Figure S6. The distribution of RFs after redundancy and OS relevancy test. For each highly correlated feature pair with r^2 greater than or equal to 0.6, the one that has a larger mean r^2 was removed. Each point represents a RF whose horizontal coordinate is the $-log_2P$ evaluated by the OS relevancy test using univariate Cox regression, and whose vertical coordinate is the mean of the square of Pearson correlation coefficient(r^2) for each feature with the rest of features. The blue dots are low-repeatable features and the pink dots are high-repeatable features. The triangular dots were the feature selected by OS relevancy test with $P<0.05$. The round dots failed the redundancy test and should be removed.

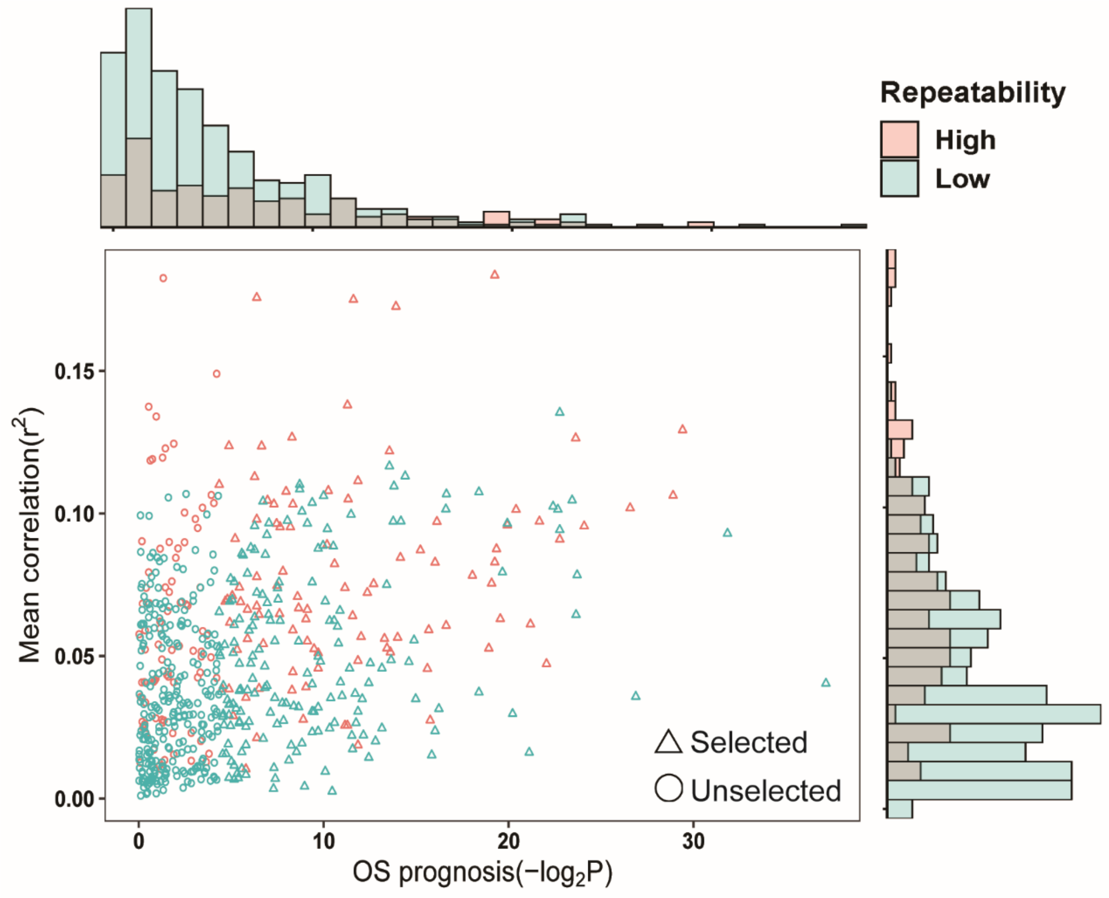

Figure S7. The process of LASSO in feature selection. Twenty-six high-repeatable RFs that were most useful to predict LRFS were selected (A, B) and listed in Table S3, and 25 low-repeatable RFs were selected (C, D) and listed in Table S4. Sixteen high-repeatable RFs that were most useful to predict OS were selected (E, F) and listed in Table S5, and 14 low-repeatable RFs were selected (G, H) and listed in Table S6.

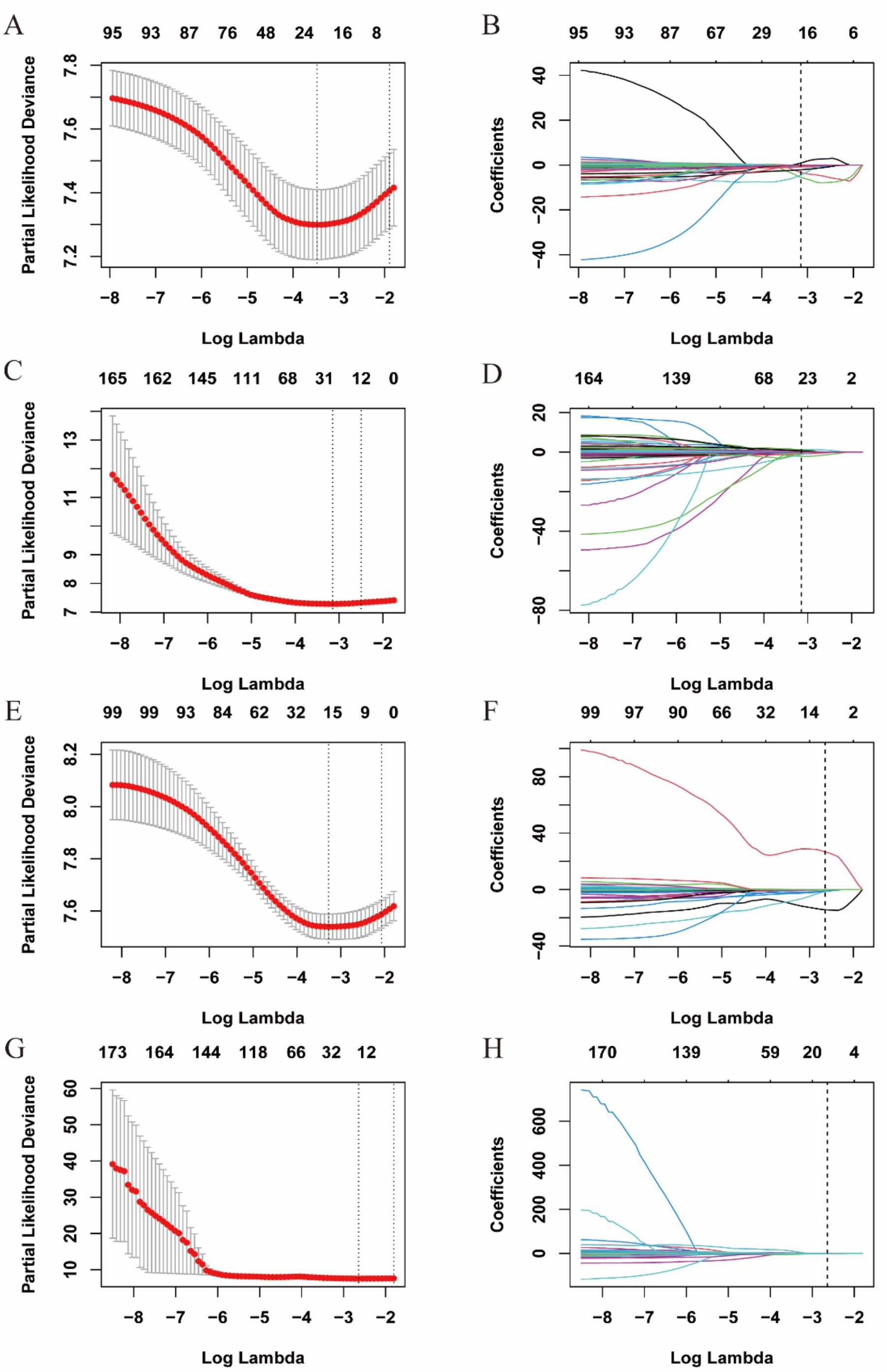
A
B
C
D
E
F
G
H
Partial Likelihood Deviance
Coefficients
Log Lambda

Figure S8. The time-dependent AUC values of the highly-repeatable features-based Cox model (High) and the lowly-repeatable features-based Cox model (Low) in training and validation sets.

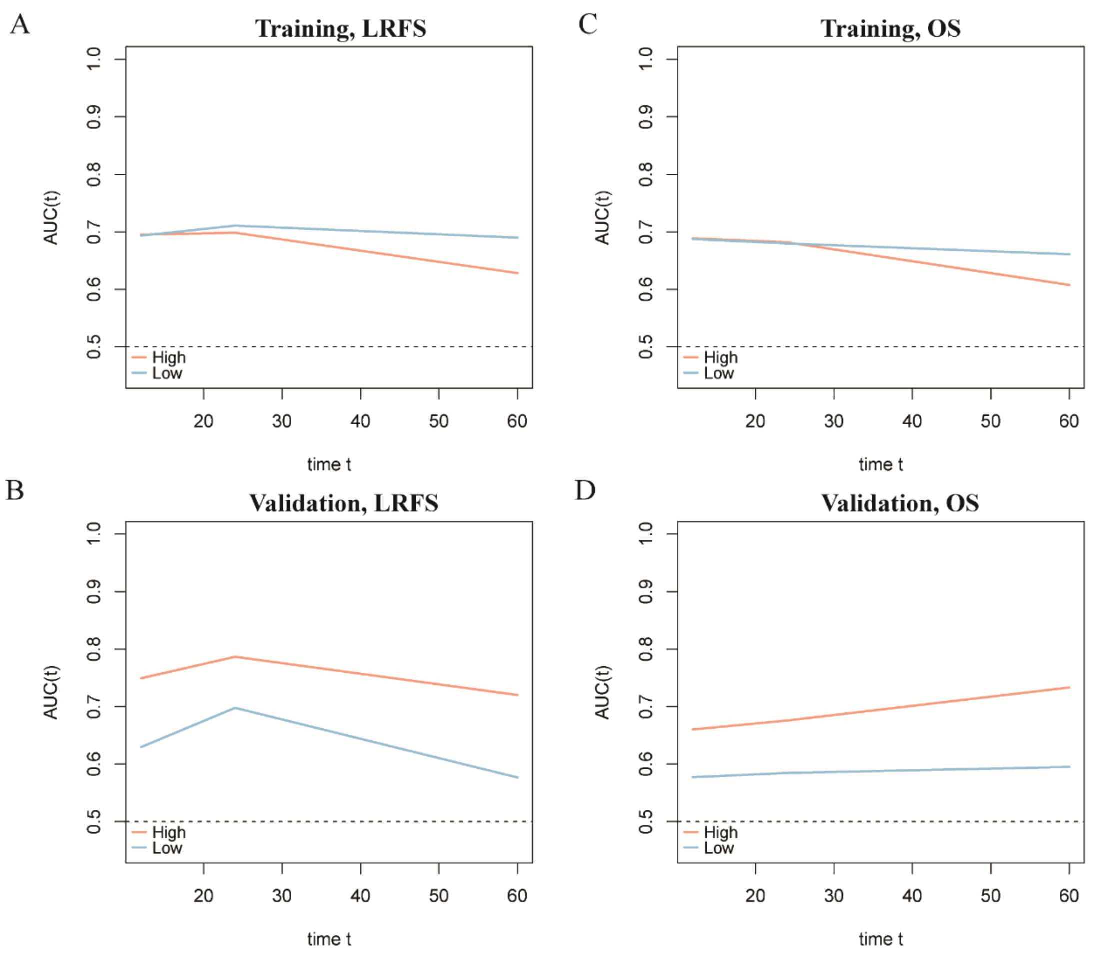

Supplemental Tables

Table S1. The Imaging acquisition parameters of scanners in two centers.

CT scanner	Xijing hospital	Sichuan hospital
Scanner model	Big Bore CT	Big Bore CT
Manufacturer	Phillips	Phillips
Tube voltage (Kv)	120	120
Tube current (mAs)	200-250	200-250
Kernel	standard	B31f
Slice thickness (mm)	5	5
Field of view（FOV） (mm^2)	350×350	350×350
Matrix	512×512	512×512
Contrast agent type	Omnipaque, GE Healthcare, USA	
Contrast agent dosage	infused 1.5 ml/kg body weight	
Contrast agent infused rate	3.0 ml/s	
Venous phase interval time	70s after injection of contrast agent	

Table S2. The parameters of image preprocessing, perturbation, and feature extraction.

Processing	Parameters	Adopted value
Preprocessing	Window width	400
	window level	40
	Resample pixel size (mm^3)	1×1×1
Perturbation	Contour randomization	randomly expand or contract by 1mm
	Rotation angles (degree)	randomly rotate with angle in [-5, 5]
Feature extraction	Image discretization bin width	8, 16, 32, 64, 128
	Image filters	Unfiltered, Laplacian-of-Gaussian (LoG,3D), Wavelet
	Kernel size of LoG filter (mm)	[1,2,3,4,5]
	Wavelet filter decompositions	[LLL, HLL, LHL, LLH, LHH, HLH, HHL, HHH]
	Feature class	First-order, GLCM, GLRLM, GLSZM, GLDM, NGTDM

Table S3. High-repeatable features selected by LASSO for predicting LRFS.

Bin width	Image filter	Feature class	Feature name	Coefficient
8	log.sigma.4.0.mm	GLCM	InverseVariance	-2.51779
8	wavelet.LLH	GLSZM	LowGrayLevelZoneEmphasis	-0.12219
16	log.sigma.1.0.mm	GLCM	Imc2	-0.03062
16	log.sigma.2.0.mm	GLRLM	ShortRunLowGrayLevelEmphasis	-0.25116
16	log.sigma.3.0.mm	GLRLM	ShortRunLowGrayLevelEmphasis	-0.33577
16	wavelet.HLL	GLCM	Imc2	-0.83651
32	log.sigma.2.0.mm	GLCM	MaximumProbability	0.070752
32	log.sigma.4.0.mm	NGTDM	Contrast	-0.5701
32	log.sigma.4.0.mm	GLSZM	GrayLevelNonUniformityNormalized	-0.09209
32	log.sigma.5.0.mm	NGTDM	Contrast	-2.54941
32	wavelet.HLL	GLSZM	GrayLevelNonUniformity	0.002708

64	log.sigma.1.0.mm	GLCM	Idn	-0.29834
64	log.sigma.5.0.mm	NGTDM	Contrast	-6.56457
64	wavelet.LLH	GLSZM	ZoneEntropy	0.004227
128	log.sigma.1.0.mm	GLRLM	RunVariance	0.010147
128	log.sigma.3.0.mm	Firstorder	Kurtosis	0.037415
128	log.sigma.4.0.mm	Firstorder	Kurtosis	0.034218
128	log.sigma.5.0.mm	GLSZM	ZoneEntropy	7.90E-06
128	log.sigma.5.0.mm	Firstorder	Skewness	-0.08684
128	log.sigma.5.0.mm	GLCM	MaximumProbability	-0.32101
128	log.sigma.5.0.mm	Firstorder	Maximum	0.000659
128	wavelet.HLL	Firstorder	Kurtosis	0.001146
128	wavelet.LHL	GLCM	JointEnergy	-1.12172
128	wavelet.LHL	GLCM	ClusterShade	-0.30622
128	wavelet.LHL	GLSZM	ZoneEntropy	0.034252
128	wavelet.LLL	NGTDM	Strength	-0.84769

Table S4. Low-repeatable features selected by LASSO for predicting LRFS.

Bin width	Image filter	Feature class	Feature name	Coefficient
8	log.sigma.1.0.mm	GLCM	MCC	-0.49233
8	wavelet.HLL	GLCM	MCC	-0.05988
8	wavelet.LHH	Firstorder	Median	-1.22464
16	log.sigma.4.0.mm	GLSZM	SmallAreaEmphasis	-0.19776
32	log.sigma.1.0.mm	NGTDM	Busyness	0.000142
32	log.sigma.4.0.mm	GLSZM	SizeZoneNonUniformityNormalized	-2.15257
32	wavelet.HHL	GLSZM	SmallAreaLowGrayLevelEmphasis	-0.02235
32	wavelet.LLH	GLRLM	RunVariance	0.031864
64	log.sigma.4.0.mm	GLRLM	ShortRunLowGrayLevelEmphasis	-0.51419
64	log.sigma.4.0.mm	GLSZM	SizeZoneNonUniformityNormalized	-0.28908
64	log.sigma.5.0.mm	GLRLM	ShortRunLowGrayLevelEmphasis	-0.2983

64	original	GLSZM	SmallAreaEmphasis	0.654167
64	wavelet.HLH	GLSZM	LowGrayLevelZoneEmphasis	0.38081
64	wavelet.LHL	GLCM	ClusterShade	-0.0075
64	wavelet.LLH	Firstorder	Skewness	-0.00847
64	wavelet.LLL	NGTDM	Strength	-0.1208
128	log.sigma.2.0.mm	GLSZM	SizeZoneNonUniformityNormalized	-0.89249
128	log.sigma.2.0.mm	GLSZM	LowGrayLevelZoneEmphasis	-0.01395
128	log.sigma.3.0.mm	GLSZM	SmallAreaLowGrayLevelEmphasis	-0.96353
128	log.sigma.4.0.mm	NGTDM	Busyness	5.47E-07
128	wavelet.HHL	GLRLM	LongRunHighGrayLevelEmphasis	0.003025
128	wavelet.LHH	GLDM	LowGrayLevelEmphasis	1.169951
128	wavelet.LHL	GLCM	InverseVariance	1.327655
128	wavelet.LLH	GLDM	DependenceNonUniformityNormalized	-0.09556

128	wavelet.LLL	NGTDM	Busyness	0.006081

Table S5. High-repeatable features selected by LASSO for predicting OS.

Bin width	Image filter	Feature class	Feature name	Coefficient
8	log.sigma.4.0.mm	GLCM	InverseVariance	-2.69055
16	log.sigma.3.0.mm	GLRLM	ShortRunLowGrayLevelEmphasis	-1.90219
16	wavelet.HLL	GLCM	Imc2	-0.46082
32	log.sigma.2.0.mm	GLCM	Idmn	28.35986
32	log.sigma.4.0.mm	NGTDM	Contrast	-11.0548
32	wavelet.HLL	GLSZM	GrayLevelNonUniformity	0.001045
64	log.sigma.5.0.mm	NGTDM	Contrast	-7.16952
128	log.sigma.1.0.mm	GLRLM	RunVariance	0.001066
128	log.sigma.3.0.mm	Firstorder	Kurtosis	0.015934
128	log.sigma.5.0.mm	Firstorder	Skewness	-0.06986
128	log.sigma.5.0.mm	GLCM	MaximumProbability	-0.17096
128	wavelet.HLL	Firstorder	Kurtosis	0.000261
128	wavelet.LHL	GLCM	JointEnergy	-0.56455
128	wavelet.LHL	GLCM	ClusterShade	-0.14997
128	wavelet.LHL	GLSZM	ZoneEntropy	0.009375

128	wavelet.LLL	NGTDM	Strength	-0.73803

Table S6. Low-repeatable features selected by LASSO for predicting OS.

Bin width	Image filter	Feature class	Feature name	Coefficient
32	log.sigma.4.0.mm	GLSZM	SizeZoneNonUniformityNormalized	-1.40633
32	wavelet.LLH	GLRLM	RunVariance	0.01792
64	log.sigma.3.0.mm	GLRLM	ShortRunLowGrayLevelEmphasis	-0.02091
64	log.sigma.4.0.mm	GLRLM	ShortRunLowGrayLevelEmphasis	-0.52047
64	log.sigma.4.0.mm	GLSZM	SizeZoneNonUniformityNormalized	-0.24801
64	log.sigma.5.0.mm	GLRLM	ShortRunLowGrayLevelEmphasis	-0.09512
64	wavelet.LLL	NGTDM	Strength	-0.03317
128	log.sigma.2.0.mm	GLSZM	SizeZoneNonUniformityNormalized	-0.56979
128	log.sigma.3.0.mm	GLSZM	SmallAreaLowGrayLevelEmphasis	-0.42204
128	original	GLRLM	ShortRunEmphasis	-0.05194
128	wavelet.HHL	GLRLM	LongRunHighGray	0.002121

			LevelEmphasis	
128	wavelet.LHH	GLRLM	ShortRunHighGrayLevelEmphasis	-0.10561
128	wavelet.LHL	GLCM	InverseVariance	0.553985
128	wavelet.LLL	NGTDM	Busyness	0.006647

Table S7. Univariate and multivariate Cox regression analyses of clinical factors associated with LRFS and OS.

Characteristics	LRFS				OS			
	Univariate analysis		Multivariable analysis		Univariate analysis		Multivariable analysis	
	HR (95%CI)	P	HR (95%CI)	P	HR (95%CI)	P	HR (95%CI)	P
Age (≥70)	1.3(1.1-1.5)	0.0067	1.2(0.97-1.4)	0.11	1.3(1.1-1.6)	0.0027	1.2(1.0-1.5)	0.0351
Gender (Female)	0.96(0.79-1.2)	0.71			1.0(0.82-1.2)	0.99		
PS (2-3)	1.2(0.98-1.4)	0.086			1.2(0.98-1.4)	0.076		
Location (Middle)	1.4(1.1-1.8)	0.0091	1.3(1.0-1.7)	0.030	1.5(1.1-1.8)	0.0039	1.4(1.1-1.9)	0.017
Location (Lower)	1.4(1.1-1.8)	0.0060	1.3(1.0-1.7)	0.031	1.5(1.1-1.8)	0.0018	1.4(1.2-1.8)	0.010
Tumor Length (≥6)	1.4(1.2-1.7)	<0.001	1.3(1.1-1.6)	<0.001	1.4(1.2-1.7)	<0.001	1.3(1.1-1.6)	0.002
T (4)	1.3(1.1-1.6)	0.002	1.4(1.1-1.7)	<0.001	1.4(1.2-1.7)	<0.001	1.4(1.2-1.8)	<0.001
N (2-3)	1.0(0.83-1.3)	0.74			1.1(0.84-1.3)	0.65		
PGTV dose (≥60)	1.1(0.96-1.4)	0.13			1.1(0.95-1.4)	0.15		

Concurrent chemotherapy (with)	0.50(0.41-0.60)	<0.001	0.51(0.42-0.63)	<0.001	0.50(0.41-0.60)	<0.001	0.54(0.43-0.66)	<0.001
